# Enhancing the retrograde axonal transport by curcumin promotes autophagic flux in N2a/APP695swe cells

**DOI:** 10.18632/aging.102235

**Published:** 2019-09-06

**Authors:** Jie Liang, Fanlin Zhou, Xiaomin Xiong, Xiong Zhang, Shijie Li, Xiaoju Li, Minna Gao, Yu Li

**Affiliations:** 1 Department of Pathology, School of Basic Medicine, Chongqing Medical University, Chongqing 400016, China; 2 Institute of Neuroscience, School of Basic Medicine, Chongqing Medical University, Chongqing 400016, China

**Keywords:** Alzheimer’s disease, curcumin, autophagy, axonal transport, dynein

## Abstract

The accumulation of autophagosomes and dysfunction at the axonal terminal of neurons play crucial roles in the genesis and development of Alzheimer’s disease (AD). Abnormalities in neuron axonal transport-related proteins prevent autophagosome maturation in AD. Curcumin, a polyphenol plant compound, has been shown to exert neuroprotective effects by increasing autophagy in AD, but the underlying mechanism of its effect on autophagy axon transport remains elusive. This study investigated the effects of curcumin on autophagosome formation and axonal transport in N2a/APP695swe cells (AD cell model) as well as the mechanism underlying those effects. Curcumin treatment significantly increased the expression of Beclin1, Atg5, and Atg16L1, induced the formation of autophagosomes, and promoted autophagosome–lysosome fusion in N2a/APP695swe cells. At the same time, curcumin promoted the expression of dynein, dynactin, and BICD2 as well as their binding to form the retrograde axonal transport molecular motor complex. Moreover, curcumin also increased the expression of the scaffolding proteins Rab7- interacting lysosomal protein (RILP) and huntingtin in N2a/APP695swe cells. Taken together, our findings indicate that curcumin increases autophagic flux by promoting interactions among autophagic axonal transport-related proteins and inducing lysosome–autophagosome fusion. This study provides evidence suggesting the potential use of curcumin as a novel treatment for AD.

## INTRODUCTION

Macroautophagy, commonly known as autophagy, is the process of intracellular self-degradation, whereby cellular components such as misfolded proteins and damaged organelles are systematically degraded and recycled [1]. Generally, autophagy occurs during the course of growth and development of the body as well as under some conditions of stress, such as starvation [2–4]. Autophagy, however, is essential in post-mitotic neurons and occurs more frequently in dendrites and distal axons, which is particularly important for maintaining cell stability [5–7]. In neurons, autophagosomes generated in distal axons can reach the cell body and bind to lysosomes through dynein-mediated retrograde axonal transport [8]. This dynamic process is called autophagic flux, and its integrity is essential for autophagy to progress [9]. Autophagosome transport generally occurs in forward and retrograde axonal transport at the same time. Forward axonal transport is mainly mediated by kinesin, whereas retrograde axonal transport is largely mediated by dynein [10]. The direction of transport entirely depends on the resultant force of forward and retrograde transport [10], which is also affected by the type of molecular motor, scaffolding proteins (Huntingtin and Rab7- interacting lysosomal protein), and adaptor proteins (Bicaudal D, two-tailed D and dynactin) recruited [11–16]. Recent studies have shown that dynein, a key protein that mediates retrograde axonal transport, often forms complexes with dynactin (dynamic actin) [17], P150Glued [18], and BICD2 (Bicaudal D, two-tailed D) [16] to guide the retrograde transport of substances. The process of autophagy retrograde axonal transport is susceptible to interference, which can lead to autophagy disorders. A variety of autophagic dysfunctions can cause neurodegeneration, such as Alzheimer’s disease (AD) [19]. Therefore, increasing autophagic activity and maintaining normal autophagic flux in neurodegenerative diseases has become a new focus of research and a therapeutic target.

Alzheimer’s disease is the most important neurodegenerative disease with a hidden onset. At present, no effective preventive or curative treatments are available [20]. The formation of intracellular neurofibrillary tangles (NFTs) and extracellular senile plaques in brain tissue are two common pathological features of AD [21]. An increasing number of studies reports autophagosome accumulation in the hippocampal and cortical axons during the early stages of AD [22, 23], which results in the blockage of autophagic flux and the inability of autophagosomes to merge properly with lysosomes around the cell body [24]. Moreover, in AD cerebrospinal fluid, the level of LAMP-2 (lysosomal-associated membrane protein 2) expression is elevated [25]. The deposition of amyloid-β (Aβ) in AD may affect retrograde axonal transport of autophagosomes [26]. Thus, AD not only hinders the neuroprotective effect of autophagy but also leads to autophagy stress and neuronal death. Together, these observations suggest that in AD, the formation of autophagosomes is unhindered, but the axonal transport of autophagosomes and the formation and fusion of autophagic lysosomes is blocked. Therefore, the search for compounds that promote the autophagy flux of AD has become the focus of current research.

Curcumin, a polyphenolic compound extracted from the turmeric rhizome, exhibits anti-inflammatory, anti-oxidant, anti-diabetic, and anti-tumor activities [27–29]. Curry has been reported to improve cognitive function in the elderly, and curcumin is the main active component of curry spices [30]. In our previous study, we found that curcumin inhibited the deposition of Aβ in the brain of APP/PS1 double transgenic AD mice, accompanied by a significant increase in the number of autophagosomes observed by electron microscopy. We also found that curcumin increased autophagic activity in AD transgenic mice. [31] In fact, few studies have investigated the mechanism whereby curcumin affects autophagosome axonal transport in AD. However, the effects of curcumin on autophagy flux and autophagy axonal transport of AD have not been further discussed. This study addresses this question by investigating whether curcumin promotes autophagy and autophagic flux in N2a/APP695swe cells by increasing axonal transport.

## RESULTS

### Curcumin induced autophagy in N2a/APP695swe cells

**Figure 1:**
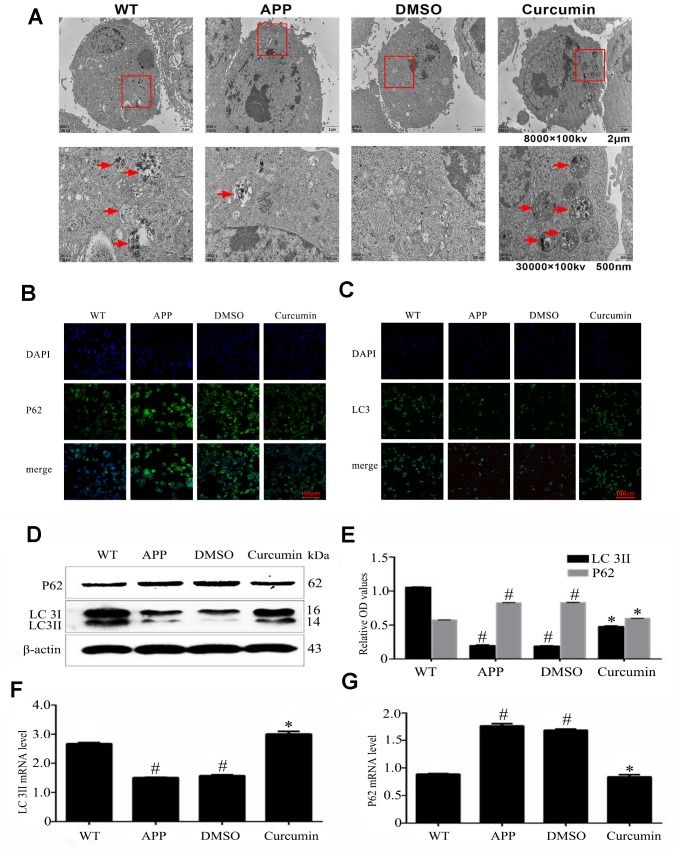
**Curcumin induced autophagy in N2a/APP695swe cells.** (**A**) Lesser and greater magnification transmission electron microscopy images for the whole cell body of each group. Arrows denote autophagic lysosomes and autophagosomes. (**B**) P62 and (**C**) LC3 immunofluorescence were used to measure the level of autophagy. Bar=100 μm. (**D**–**E**) Western blot analysis of LC3II, and p62 in each group. (**F**–**G**) Relative mRNA expression of (**F**) LC3 and (**G**) P62 of each group; The data represent as mean ± SEM of a typical series of 3 experiments (# P<0.05, compared to the WT group; * P<0.01, compared to the APP or DMSO group).

TEM (Transmission electron microscopy) images revealed that autophagic structures were clearly present in the WT group (red arrows), whereas few were observed in the N2a/APP695swe and DMSO groups (Figure 1A). After curcumin treatment for 24 hours, the number of autophagy-like structures significantly increased; all autophagic structures were found to contain damaged organelles.

LC3 (microtubule-associated protein 1 light chain 3 [MAP1A/1BLC3]) is a marker for autophagy, and P62 (sequestosome 1) is the substrate of autophagy degradation. LC3 and P62 were present in the cytoplasm but not the nucleus (Figure 1B, 1C). Compared to the WT group, the expression of LC3 was lower in the N2a/APP695swe and DMSO groups (P < 0.05). Curcumin treatment markedly increased LC3 expression, as indicated by increased fluorescence intensity (P < 0.05). In contrast, the expression of P62 was much greater in the N2a/aAPP695swe group and DMSO group than in the WT group (P < 0.05), and curcumin notably decreased P62 expression (P < 0.05). The expression of LC3 and P62 at the mRNA and protein levels were in accordance with the IF results (Figure 1D–1G).

### Curcumin enhanced the expression of autophagy-related genes encoding proteins Beclin1, Atg5, and Atg16L1 in N2a/APP695swe cells

**Figure 2:**
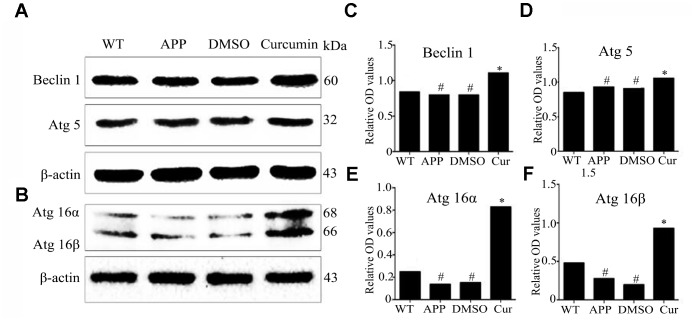
**Curcumin enhanced the expression of autophagy-related genes encoding proteins, Beclin1, Atg5, and Atg16L1 in N2a/APP695swe cells.** (**A**–**F**) Western blot analysis of Beclin1, Atg5, Atg16α and Atg16β in each group; Representative blot (**A**–**B**) and summary graph of densitometric analysis (**C**–**F**); The data represent as mean ± SEM of a typical series of 3 experiments (# P<0.05, compared to the WT group; * P<0.01, compared to the APP or DMSO group).

Further autophagy-related proteins detected in this experiment include Beclin1, Atg5, and Atg16L1. The protein expression of Beclin1 was significantly lower in the APP and DMSO groups (P < 0.05) than in the WT group, but no significant difference was observed between the DMSO and APP groups (P > 0.05) (Figure 2A, 2C). Curcumin clearly increased the expression of Beclin1 (P < 0.001). The optical density values indicating Atg5 expression were similar in the AP and DMSO groups (P > 0.05), and Atg5 expression was slightly lower in the WT group than that in the APP group (P < 0.05) and higher in the curcumin group (P < 0.001) (Figure 2A, 2D). No significant difference in the expression levels of Atg16L1α and β subtypes was observed between the APP and DMSO groups (P > 0.05). Atg16L1-α and -β expression were clearly higher after curcumin treatment (P < 0.001) (Figure 2B, 2E, and 2F).

### Curcumin enhanced autophagic flux and autophagosome-lysosome binding in N2a/APP695swe cells

**Figure 3:**
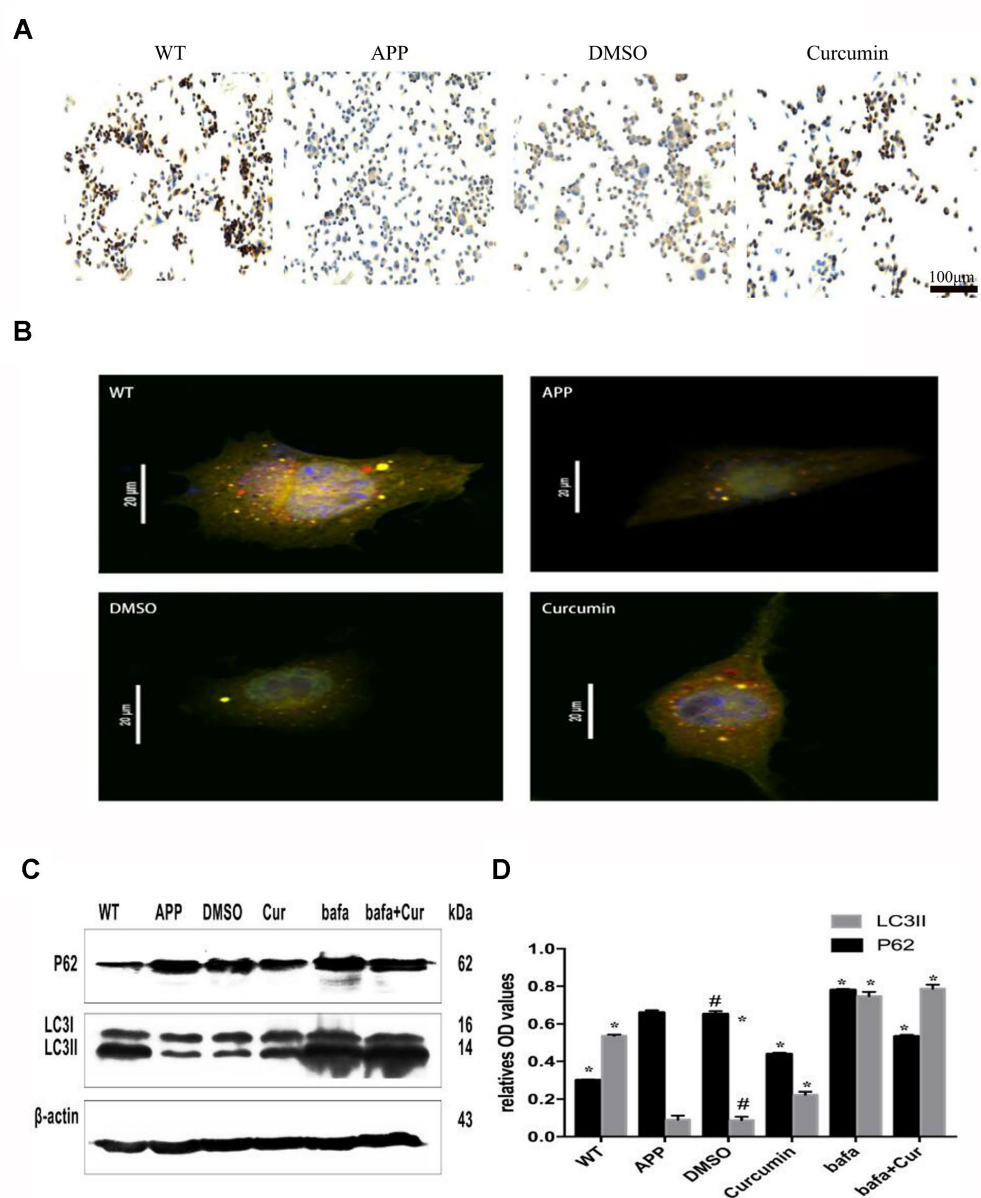
**Curcumin enhanced autophagic flux and autophagosome-lysosome binding in N2a/APP695swe cells.** (**A**) LAMP2 immunocytochemistry was used to detect the expression of lysosome in each group. Bar = 100 μm. (**B**) Fluorescent microscopy analysis of cells transiently overexpressing mRFP-GFP-LC3.Bar=20 μm. (**C**–**D**) Western blot analysis of P62 and LC3II in each group; Representative blot (**A**) and summary graph of densitometric analysis (**D**); The data represent as mean ± SEM of a typical series of 3 experiments (# P<0.05, compared to the WT group; * P<0.01, compared to the APP or DMSO group; **, P<0.01, compared to the APP group or DMSO group; ##, P<0.01, compared to the Bafa group).

The expression of lysosomal-associated membrane protein 2 (LAMP2), a protein involved in lysosome function, was significantly lower in the APP and DMSO groups as compared to the WT group (P < 0.05) (Figure 3A). Curcumin treatment increased LAMP2 expression (P < 0.01).

Furthermore, N2a/APP695swe cells were transfected with double-labeled autophagic adenovirus (mRFP-GFP-LC3), and the number of autophagosomes and lysosomes were determined after the treatment. As shown in Figure 3B, both yellow (autophagosomes) and red (lysosome) fluorescent signals were present in the cytoplasm of the WT and curcumin groups, but only a small amount of red and yellow fluorescent signals was observed in the APP group. Fluorescence in the DMSO group was similar to that of APP group.

In order to further explore whether curcumin promotes the fusion of autophagosome and lysosome, we used the lysosomal inhibitor Bafilomycin A1 (Bafa) to treat N2a/APP695swe cells. After treatment with Bafilomycin A1 (Bafa), the expression of P62 and LC3II in the lysates of each group was measured by western blot (Figure 3C, 3D). The expression of LC3II was much lower in the APP group and DMSO groups (P < 0.01) than in the WT group. After curcumin or Bafa treatment, the expression of LC3II increased markedly (P < 0.01). The combination of curcumin and Bafa also increased the expression of LC3II protein. P62 expression was much higher in the APP and DMSO groups than in the WT group. P62 expression was decreased by curcumin treatment but significantly increased by Bafa treatment, reaching expression levels higher than that of the APP group. Combined treatment with curcumin and Bafa resulted in P62 expression levels in-between those of the single treatments.

### Curcumin increased the expression of retrograde axonal transport molecular motor and scaffolding proteins, decreased the expression of forward axonal transport molecular motor and promoted autophagy flux in N2a/APP695swe cells

**Figure 4:**
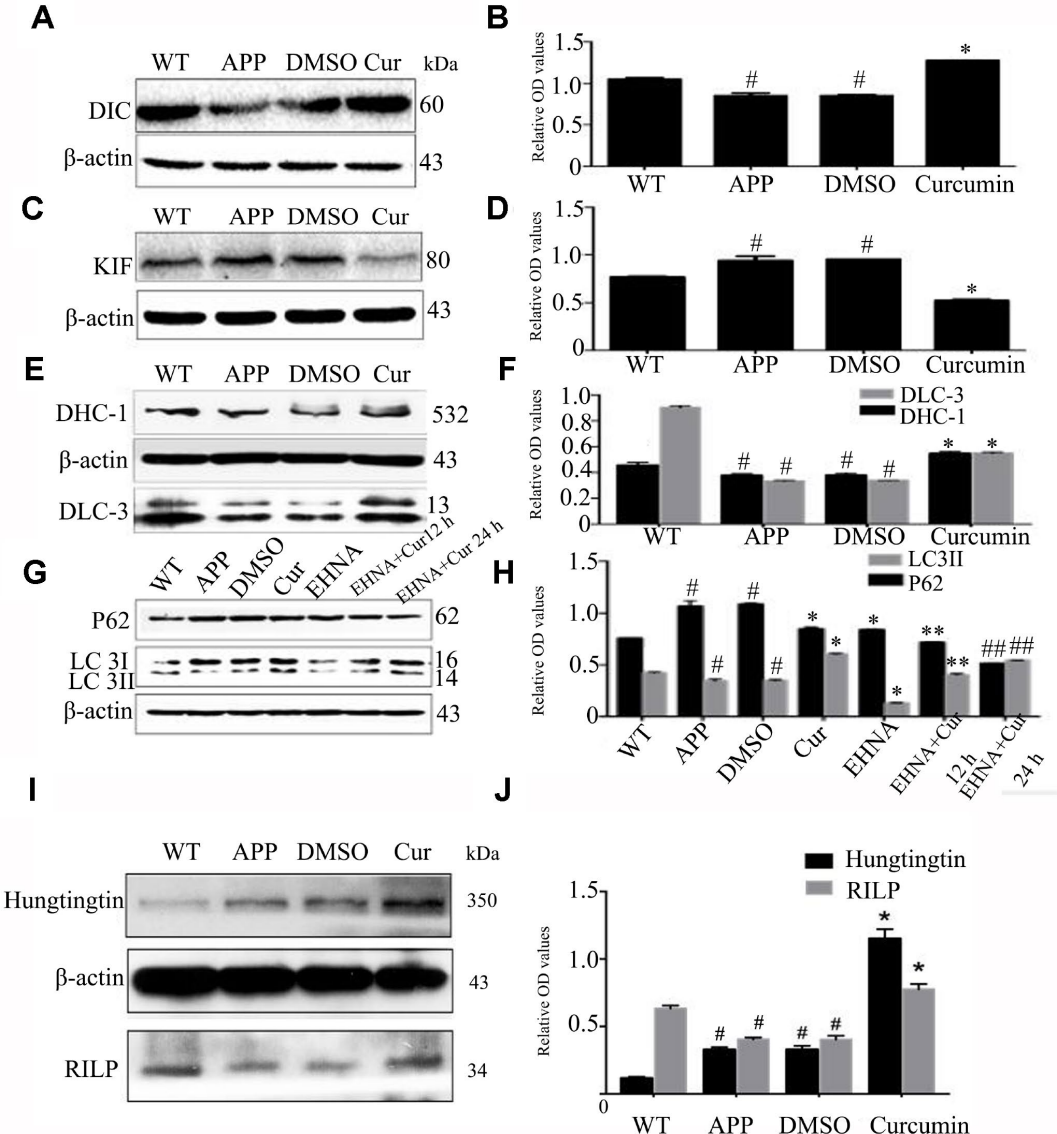
**Curcumin increased the expression of retrograde axonal transport molecular motor and scaffolding proteins, decreased the expression of forward axonal transport molecular motor and promoted autophagy flux in N2a/APP695swe cells.** (**A**–**F**) Western blot analysis of retrograde axonal transport molecular motor DIC (**A**–**B**), DHC1, DLC-3 (**C**–**D**) and forward axonal transport molecular motor KIF (**E**-**F**) in each group; The data represent as mean ± SEM of a typical series of 3 experiments (# P<0.05, compared to the WT group; * P<0.01, compared to the APP or DMSO group). (**G**–**H**) Western blot analysis of P62 and LC3II in each group with(out) dynein inhibitor EHNA; The data represent as mean ± SEM of a typical series of 3 experiments (# P<0.05, compared to the WT group; * P<0.01, compared to the APP or DMSO group; **, P<0.01, compared to the EHNA group; ##, P<0.01, compared to the EHNA group). (**I**–**J**) Western blot analysis of scaffolding proteins Huntingtin and RILP in each group; The data represent as mean ± SEM of a typical series of 3 experiments (# P<0.01, compared to the WT group; * P<0.001, compared to the APP or DMSO group).

Expression of retrograde axonal transport molecular motor, DIC (the dynein intermediate chain) was markedly lower in the APP and DMSO groups than in the WT group (P < 0.05) (Figure 4A, 4B). After curcumin treatment, DIC expression increased significantly (P < 0.05), and the expression of forward axonal transport molecular motor, KIF (kinesin family member), was notably lower than that in the APP and DMSO groups (P < 0.05) (Figure 4C, 4D). Relative to WT, curcumin significantly decreased KIF expression (P < 0.01). Next, Expression of the retrograde axonal transport molecule motor, DHC-1(dynein cytoplasmic 1 heavy chain 1) and DLC-3(dynein light chainTctex-type3) in N2a/APP695swe cells were examined. No significant difference was observed in the expression of DHC-1 or DLC-3 between the APP and DMSO groups (P > 0.05) (Figure 4E, 4F). Curcumin treatment significantly increased the expression of both of these proteins (P < 0.01).

In order to further study, this study used dynein inhibitor Erythro-9-(2-hydroxy-3-nonyl) adenine hydrochloride (EHNA) to inhibit dynein. After treatment of N2a/APP695swe cells with the EHNA, with or without curcumin, the expression of LC3II was lower and P62 higher in the APP (P < 0.05) and DMSO (P < 0.05) groups as compared to the WT group (Figure 4G, 4H). Curcumin treatment markedly increased the expression of LC3II and decreased the expression of P62 (P < 0.05). Interestingly, EHNA treatment alone also lowered the expression of P62 and LC3II. Combined treatment with EHNA and curcumin further lowered the expression of P62 but increased the expression of LC3II (P < 0.01).

During retrograde axonal transport, the molecular motor dynein also requires the assistance of scaffold proteins, including Huntingtin and Rab7-interacting lysosomal protein (RILP). In western blot analysis, we observed no difference in the expression of Huntingtin and RILP between the DMSO and APP groups (P > 0.05) (Figure 4I, 4J); The expression of Huntingtin was lower and that of RILP was higher than in the WT group (P < 0.05). Curcumin treatment significantly increased the expression of Huntingtin and RILP (P < 0.001).

### Curcumin promoted the expression of binding proteins dynactin (P150 and P50) and BICD2 (Bicaudal D, two-tailed D) in N2a/APP695swe cells

**Figure 5:**
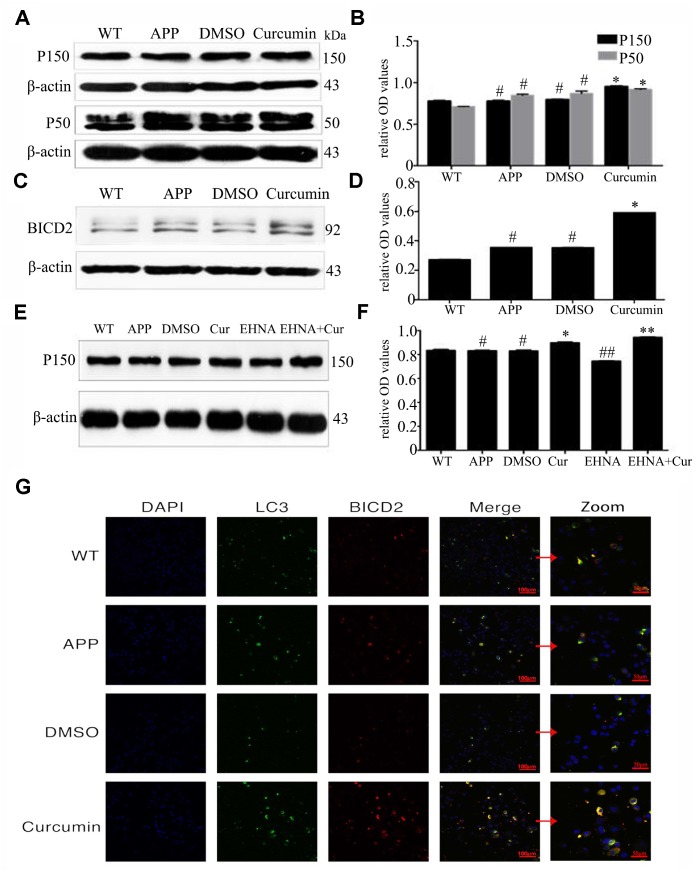
**Curcumin promoted the expression of binding proteins dynactin (P150 and P50) and BICD2 (Bicaudal D, two-tailed D) in N2a/APP695swe cells.** (**A**–**D**) Western blot analysis P150, P50 and BICD2 in each group; The data represent as mean ± SEM of a typical series of 3 experiments (* P<0.01, compared to the APP or DMSO group). (**E**–**F**) Western blot analysis of P150 in each group with(out) dynein inhibitor EHNA; The data represent as mean ± SEM of a typical series of 3 experiments (* P<0.01, compared to the APP or DMSO group; **, P<0.01, compared to the EHNA group; ##, P<0.01, compared to the APP or DMSO group). (**G**) Confocal fluorescence microscopy showed the expression of BICD2 and LC3 in each group. Bar=100/50 μm.

DIC binds to the microtubule-associated protein dynactin to form a functional complex required for reverse axonal transport. P150 and P50 are two forms of dynactin, and BICD2 is a connector protein that joins dynein to dynactin in a ternary complex. We observed that the level of P150 expression did not differ between the DMSO, APP, and WT groups (P > 0.05) (Figure 5A, 5B). Curcumin treatment significantly increased P150 expression (P < 0.05). P50 expression was higher in the APP and DMSO groups than that in the WT group (P < 0.05), and curcumin treatment increased its expression (P < 0.05). The expression of BICD2 was slightly higher in the DMSO group than in the APP group (P < 0.05), and curcumin markedly increased BICD2 expression (P < 0.001) (Figure 5C, 5D). Treatment with the motor protein inhibitor EHNA significantly decreased the expression of P150, and curcumin treatment increased P150 expression (P < 0.01) (Figure 5E, 5F).

Confocal fluorescence microscopy showed that BICD2 and LC3 were expressed in the cytoplasm but not in the nucleus and that both proteins were expressed in all groups (Figure 5G). No colocalization of these proteins was observed in any group. Curcumin treatment resulted in colocalization signals in some of the cells. Therefore, curcumin may promote interaction between BICD2 and LC3 and increase retrograde axonal transport of autophagosomes.

## DISCUSSION

In AD, aging neurons exhibit gradually decreasing the function of the protein hydrolysis process. At the same time, the dependence of neuron survival on the clearance of the damaged proteins increases [19]. An appropriate increase in autophagy may maintain the dynamic balance of processes in neurons and reduce the pathological damage of AD. In the APP transgenic mouse model, rapamycin treatment ameliorates cognitive deficits and reduces pathological damage caused by defective tau protein by increasing autophagy [32, 33]. Wang et al. report that curcumin induces autophagy and ameliorates hippocampal neuron damage induced by lithium-pilocarpine in a rat model of status epilepticus [34]. Jaroonwitchawan et al. found that curcumin attenuates paraquat-induced death of SH-SY5Y cells by increasing autophagy and decreasing the accumulation of APP [35]. Our previous study reports that in the APP/PS1 double transgenic AD mouse model, curcumin decreases the production of Aβ and improves cognitive dysfunction by activating autophagy through regulation of the PI3K/Akt pathway [31]. In fact, autophagy is well known as a double-edged sword. On the one hand, it plays neuroprotective roles in an AD model *in vivo* via clearing away its harmful substances; on the other hand, aggregation of autophagosomes can lead to autophagic stress, causing neuron damage [36]. Our previous studies report that curcumin treatment increases autophagy and neuron health, but how curcumin increases autophagy without causing simultaneous autophagic stress was unclear. This question led to the present study.

Evidence that curcumin promotes the formation of autophagy lysosomes comes from TEM images (Figure 1A). Meanwhile, after treating N2a/APP695swe cells with curcumin, we found an increase in the number of autophagy-like structures. Immunofluorescence and western blot of LC3 and p62 also supported this phenomenon. In the process of autophagy formation, LC3 is the most characteristic mammalian core autophagy protein, LC3I exists in the cytoplasm, binding with phosphatidylethanolamine to form LC3II, binding to autophagy membrane, which can be used as a marker for autophagy [37]. SQSTM 1/p62 is a selective autophagy receptor that isolates ubiquitin proteins into autophagy by interacting with LC3 [38]. In addition, p62 is a substrate for autophagy degradation, so its degradation can be used as a marker for autophagy clearance [38, 39]. In this study, we found that the expression of LC3II increased and p62 decreased after curcumin treatment (Figure 1B–1G).

At different stages of autophagosome formation, in addition to LC3 and p62, 16 autophagy-related proteins are involved in autophagy induction [40, 41]. Beclin1–PI3KC3 complex formation is induced by autophagy and plays an important role in autophagosome nucleation and initial membrane formation in phagocytic cells [42, 43]. Beclin1 expression was found to decrease in damaged brain regions (the entorhinal cortex and hippocampus) during the early stage of AD, decreasing simultaneously with the development of cognitive impairment [44]. In an AD mouse model, beclin1 knockout mice had elevated levels of Aβ and impaired autophagy clearance [43]. Thus, increasing the expression of beclin1 might have a therapeutic effect on AD. In our previous study, we observed that curcumin increased the expression of beclin1 in the CA1 region in AD transgenic mice [31]. Studies have shown that Atg5 and beclin1 are associated with Aβ clearance in AD. The Atg12-Atg5-Atg16 complex forms during autophagosome formation, promoting autophagy [45, 46]. Our results are similar, curcumin enhanced the expression of autophagy-related genes encoding proteins Beclin1, Atg5, and Atg16L1 in N2a/APP695swe cells (Figure 2).

The accumulation of misfolded proteins such as that of Aβ in AD is a key characteristic of many neurodegenerative diseases. Studies have shown that the autophagic-lysosomal pathway is a key regulator of Aβ production and clearance, and its dysfunction is considered a key cause of abnormal Aβ accumulation in AD brain. In addition, Lin and Nixon et al. [47, 48] have shown that autophagic vesicles containing excessively phosphorylated tau protein accumulate in a mouse model of AD. Induction of autophagy can promote the degradation of insoluble tau protein in a triple transgenic mouse model of AD [32]. Therefore, the up-regulation of autophagy and the patency of autophagy in AD can further inhibit the neurotoxicity induced by Aβ and improve the pathological damage caused by misfolded tau protein [49]. Thus, according to the above result, the ability of curcumin to increase autophagy and improve autophagic flux may serve as a new target for the treatment and prevention of AD.

In the process of inducing autophagy, curcumin not only promotes the production of autophagy, but also plays an active role in the process of autophagy and lysosome. LAMP1 and LAMP2 proteins are essential for lysosome–autophagosomes fusion [50, 51]. In AD cerebrospinal fluid, the level of LAMP-2 expression is elevated [25]. In the present study, we observed that curcumin increased the expression of LAMP-2 and increased the binding between autophagosomes and lysosomes (Figure 3A–3B). Furthermore, the lysosomal inhibitor Bafa significantly increased the expression of LC3II and P62, suggesting that bafilomycin hindered lysosome–autophagosome fusion, causing excess accumulation of autophagosomes. Combined treatment with Bafa and curcumin increased the expression of LC 3II but decreased the expression of P62, suggesting that autophagic flux was restored (Figure 3C–3D). The above results showed that curcumin increased the number of lysosomes and promoted their fusion with the increased numbers of autophagosomes, thus promoting their degradation.

Autophagosomes form in the synaptic domain and are then successfully transported by dynein–dynactin along the microtubules to the soma for fusion with lysosomes. Studies conducted in HeLa cells have found that mature autophagosomes aggregate around the centrosome, and the dynein–dynactin complex drives retrograde axon movement, promoting its fusion with lysosomes [52, 53]. In dynein knock-down experiments, Jahreiss et al. observed that autophagosome–lysosome fusion requires dynein-mediated retrograde axonal transport [52]. Defects in autophagy clearance in AD were originally proposed by Nixon [47] and Boland [23], who observed that autophagosomes contain undigested materials and accumulate in neurons. Tammineni et al. [54] found that dynein-mediated retrograde transport was impaired in the brains of AD mice, and autophagosomes accumulated in large numbers along dystrophic neurites. In the present study, we observed that curcumin increased the expression of DIC, DHC1, DLC-3 and decreased the expression of KIF (Figure 4A–4F), suggesting that curcumin promotes retrograde transport and inhibits antegrade transport of autophagosomes in AD model N2a/APP695swe cells.

Dynein-driven retrograde axonal transport also requires the assistance of scaffolding proteins that regulate the activity of relevant molecules to control the direction of transport. Common scaffold proteins that assist in retrograde axonal transport include RILP and Huntingtin [11, 12]. Studies have shown that the combination of Huntingtin, dynein, and dynactin increases the stability of active motor complexes, thereby promoting sustained motility [17]. In N2a/APP695swe cells, we observed high Huntingtin and low RILP expression (Figure 4I–4J). Curcumin increased the expression of both Huntingtin and RILP; thus, curcumin has a synergistic effect on regulating the retrograde axonal movement of the dynein complex.

Our above results show that curcumin increased the expression of dynein heavy chain and light chain. Treatment with the dynein inhibitor EHNA hindered the autophagic flux; simultaneous curcumin treatment ameliorated the inhibitory effects of EHNA to some extent. Dynactin is composed of more than 20 subunits; its largest subunit, P150, interacts with microtubules and dynein motor protein complexes. The CC1 and CC2 domains of P150 mediate bind to DIC and other dynactin subunits [55]. P150 is also considered to be a key player in linking various substances to the dynein complex and then to the soma [17]. In the present study, the two forms of dynactin protein, P50 and P150, and BICD2 increased after curcumin treatment (Figure 5A–5D). After treatment with EHNA, the expression of P150 decreased, and curcumin treatment counteracted the effect of EHNA (Figure 5E–5F). Curcumin also promoted the co-localization of BICD2 and LC3 (Figure 5G). The observation suggests that curcumin regulates autophagosome retrograde axonal transport and autophagic flux by modulating the dynein–dynactin–BICD2 complex.

**Figure 6:**
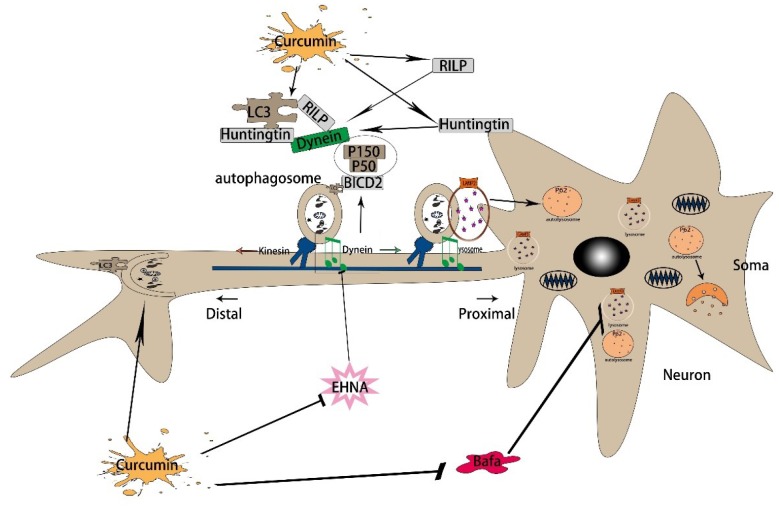
**The mechanism of curcumin promoting autophagic flux in AD.** First, curcumin induces autophagy by regulating Beclin1, Atg5, and Atg16. Then, curcumin promotes the retrograde axonal transport of autophagy by increasing the expression of dynein, Huntingtin, and RILP. By the time, it increases the dynactin and BICD2, also promoting the binding of BICD2 and LC3. Finally, it promotes the binding of autophagy corpuscles and lysosomes, thus enhances autophagy flux.

In conclusion, our findings suggest that curcumin increases autophagy and lysosome–autophagy fusion in N2a/APP695swe cells and increases the expression and interactions of autophagic axonal transport-related proteins, thereby facilitating the axonal transport of autophagosomes and autophagic flux. Our study provides new insights into the mechanism whereby curcumin promotes autophagic flux in AD, suggesting a new strategy for the treatment of AD (Figure 6).

In the course of our study, we found that curcumin has an inhibitory effect on forward axonal transport, but it has not been further studied because of objective reasons. Therefore, in future research, we plan to further explore the upstream target of curcumin action on autophagosome axonal transport by perturbing the expression of one or more genes. Meanwhile, the effect of excessive tau phosphorylation on autophagosome axonal transport in AD was included in future research. In addition, we will continue to explore the effects of curcumin on autophagic flux and autophagosome axonal transport *in vivo* or in another cell model to provide new insights and evidence for its use in the treatment of AD.

## MATERIALS AND METHODS

### Cell culture

Mouse neuroblastoma cell line N2a/WT cells and N2a / APP695swe cells were from Obio Technology (Shanghai, China). N2a/WT cells were cultured in the solution of 79% Opti-MEM I Reduced-Serum Medium (opti-MEM, Gibco), 20% fetal bovine serum (Hyclone), 1% solution of penicillin and streptomycin (Beyotime). Compared with the N2a/WT cells, N2a/APP695swe cells were additionally supplemented with 0.04% puromycin (5 mg/ml) to screen for cells stably expressing the APP695swe gene. They were maintained in the incubator containing 5% CO2 at 37°C.

### Medicine treatment

Curcumin was bought from Sigma-Aldrich. Also EHNA (erythro-9-(2-hydroxy-3-nonyl) adenine hydrochloride) and Bafa (bafilomycin A1)was purchased from Selleck Chemicals. The cells were plated onto 6-well plates at a density of 1×10^6^ cells/ml. Then, N2a/APP695swe cells were treated with the dynein ATPase inhibitor EHNA or bafilomycin A1 at a final concentration of 100 nmol/l and 10nmol/l. Also, 5 mmol/l curcumin was used to treat N2a/APP695swe cells for 24 hours. After 24 hours of EHNA, bafilomycin A1 and curcumin treatment, cells were subjected to Western blot analyses and immunocytochemical analysis.

### Antibodies

The mouse anti-APP, mouse anti-KIF3B and rabbit anti-ULK1 were purchased from Cell Signaling. Rabbit anti-Atg16L1 and rabbit anti-Atg5 were purchased from ABclonal (China). Rabbit anti-β-actin, rabbit anti-LC3B-Specific, rabbit anti-P50, rabbit anti-DHC1, rabbit anti-DlC3 and rabbit anti-P62/SQSTM1 were purchased from Proteintech (US). Mouse anti-DIC was bought from Millipore. Rabbit anti-RILP and rabbit anti-Lamp2 were purchased from Abcam. Mouse anti-Huntingtin and mouse anti-BICD2 were purchased from Santa Cruz. All the secondary antibodies were purchased from Bioworld Technology.

### Transmission electron microscopy

For transmission electron microscopy detection, the N2a/APP695swe cells were cultured in 6-well plates and the concentration is greater than 1×106 cells/well at least. The cells were harvested at 24hours after treat with curcumin. Then 0.1% trypsin-EDTA buffer was used to digest cells, and the cell lysis was centrifuged at 800*g for 5 minutes. Phosphate buffer saline (PBS, PH7.4) was used to resuspend cells before they were centrifuged at 1200g for 10 minutes. Furthermore, the cell pellets were fixed in 2.5% electron microscopy-specialized glutaraldehyde for 2 hours, washed several times with PBS (0.01 M), stained with 1% osmium tetroxide for 2 hours, and then dehydrated in a gradient series of alcohol solutions. The samples were placed in propylene oxide, embedded in the epoxy resin Epon812, and cut into ultrathin sections. After uranyl acetate and lead citrate double staining, cells were observed by a transmission electron microscopy of Philips EM208S.

### Transfection assay and adenoviral tandem fluorescent-tagged LC3 (mRFP-GFP-LC3) analysis

The day before transfection, cells were seeded into 24-well plates and cultured overnight. The next day, and at a cell density of between 50–70%, viral infection was performed. The adenoviral mRFP-GFP-LC3 was removed from the -80°C freezer in advance and thawed on ice. Transfection steps were performed in strict accordance with the manufacturer’s instructions. Cells were transfected with adenoviral mRFP-GFP-LC3 at a multiplicity of infection (MOI) value of 50. The medium was refreshed 6 h later and the cells were cultured for a further 24 hours. Transfected cells were treated with medicines for 24 hours. Cells were then fixed with 4% paraformaldehyde and viewed under a laser scanning confocal microscope. The yellow spots which appeared after merge indicated autophagosomes, the red spots indicated autolysosomes. The intensity of autophagic change could be determined by counting the different colored fluorescent dots on confocal microscopy. We use an artificial counting method, and at least 50 cells were counted in each experiment.

### Immunofluorescence

After treatment, the cell slides were washed three times with PBS and fixed with 4% paraformaldehyde for 15 minutes and then permeabilized with 0.2% Triton X-100 for 10 minutes at room temperature. Normal goat serum was added to the slides and incubated for 30 minutes at room temperature. The primary antibody was then added and placed in a humid box overnight at 4°C. The next morning, the slides were washed 3 times with PBST and incubated with Dylight 549, Goat Anti-Mouse IgG(Abbkine, A23310) or Dylight 488, Goat Anti-Rabbit IgG (Abbkine, A23220) at 37°C for 1h, and the slides were stained with the blue DNA fluorescent stain 4’, 6-diamidino-2-phenylindole (DAPI) for 5 minutes. Finally, the slides were sealed with a mounting medium containing anti-fluorescence quencher, and observed under a fluorescence microscope.

### Immunohistochemistry

After treatment, the cell slides were washed three times with PBS and fixed with 4% paraformaldehyde for 15 minutes and then permeabilized with 0.2% Triton X-100 for 10 minutes at room temperature. Normal goat serum was added to the slides and incubated for 30 minutes at room temperature. The staining protocol employed a modified streptavidin-HRP immunohistochemistry procedure (CoWin Century Biotechnology, Inc, China). Briefly, the primary antibody was then added and placed in a humid box overnight at 4°C. The next morning, the slides were washed 3 times with PBST and treated with peroxidase-conjugated streptavidin and visualized by the diaminobenzidine (DAB) Kit (CoWin Century Biotechnology, Inc, China), and sections were counterstained with haematoxylin. Finally, the slides were sealed with neutral gum and observed under Nikon optical microscope.

### RNA isolation and quantitative real-time PCR analysis

Total RNA was extracted from cultured cells using RNAiso Plus (TaKaRa) according to the manufacturer’s instructions. For mRNA expression analysis, the synthesis of cDNA was conducted with 1 ug of total RNA using PrimeSriptTM RT reagent Kit (TaKaRa) and gene expression quantified using SYBR Premix Ex TaqTM II (TaKaRa). All reactions were performed in triplicate. Primer Sequences used: LC3B-F:5′-ATC ATC CTG GGG GAC TCT GG-3′; LC3B-R: 5′-ATT GCT GTC CCG AAT GTC TC-3; P62-F: 5-CCG TCT ACA GGT GAA CTC CAG TCC-3′; P62-R: 5′-AGC CAG CCG CCT TCA TCA GAG-3′; β-actin-F: 5′-GGA AAT CGT GCG TGA CAT C-3′; β-actin-R: 5′,-CCA AAA GAA GCT GGA A-3′.

### Western blot assay

The cells were harvested in RIPA buffer (Beyotime, China). Protein concentration was determined by BCA Protein Assay Kit (Beyotime, China) at 570 nm. Equal amounts of protein were loaded in each lane for SDS-PAGE Bis-tris gel and then transferred to polyvinylidene difluoride membranes (Millipore, Billerica, MS,USA). The membranes were washed with blotting buffer (Tris-buffered saline containing 0.1% Tween-20) and then blocked for 120 minutes in the buffer containing 5% non-fat powdered milk. After washed 3 times with blotting buffer, the membrane was incubated at 4°C overnight with primary antibody. After further washing in blotting buffer, the membrane was incubated with secondary antibody at room temperature for 40 minutes. Last, the membranes were developed with ECL Western Blotting Detection Reagents, and Image J was used to quantitate the expression of proteins.

### Statistical analysis

Statistical analysis was performed using SPSS 18.0 software. All the data were presented as means ± S.E.M. And data were statistically analyzed by one-way ANOVA, followed by Bonferroni post hoc test, or were analyzed with Student’s t test. P < 0.05 was considered to be statistical significance.
